# Supplementary material for: Additional flight delays and magnetospheric–ionospheric disturbances during solar storms
Source: Sci Rep. 2023 Feb 24;13:3246. doi: 10.1038/s41598-023-30424-2 (PMC9951832; doi:10.1038/s41598-023-30424-2)
Supplement: Supplementary file 1 — Supplementary Information. [file 41598_2023_30424_MOESM1_ESM.pdf]

Supplementary Material

Appendix

The list of the SWEs is collected from below:

SFs:
https://www.ngdc.noaa.gov/stp/space-weather/solar-data/solar-features/solar-flares/x-rays/goes/xrs/

CMEs:
https://izw1.caltech.edu/ACE/ASC/DATA/level3/icmetable2.htm

SEPs:
https://umbra.nascom.nasa.gov/SEP/

All the selected SWEs are listed in Table A.

Table A. Lists of all SWEs

SWE No.	Event Type	Start Date (YearMonthDay)	Start Time (HourMinute UT)
1	CME	20150107	0700
2	SF	20150113	0413
3	SF	20150114	1230
4	SF	20150122	0443
5	SF	20150126	1646
6	SF	20150128	0421
7	SF	20150204	0208
8	SF	20150209	2219
9	SF	20150302	0631
10	SF	20150305	1706
11	SF	20150307	2159
12	SF	20150309	1422
13	SF	20150315	2242
14	CME	20150322	0200
15	CME	20150331	1800
16	SF	20150408	1437
17	SF	20150421	0708
18	SF	20150423	0918
19	SF	20150505	0942
20	CME	20150518	2000
21	SF	20150611	0849
22	SF	20150613	0720
23	SF	20150618	0033
24	SEP	20150618	1135
25	SF	20150620	0628
26	SEP	20150621	2135
27	SF	20150625	0802
28	SEP	20150626	0350

29	SF	20150703	1247
30	SF	20150706	0824
31	CME	20150713	0600
32	CME	20150807	1600
33	CME	20150815	2100
34	SF	20150821	0156
35	SF	20150824	0726
36	SF	20150828	1304
37	SF	20150830	0201
38	CME	20150908	0000
39	CME	20150913	0700
40	SF	20150917	0804
41	SF	20150920	0455
42	SF	20150927	1020
43	SF	20151001	1303
44	SF	20151004	0234
45	SF	20151015	22:45
46	SF	20151017	2009
47	CME	20151025	1400
48	SEP	20151029	0550
49	SF	20151031	1748
50	SF	20151104	0320
51	CME	20151107	0600
52	SF	20151224	0149
53	SF	20151228	1120
54	CME	20151231	1700
55	SEP	20160102	0430
56	CME	20160119	1000
57	CME	20160124	1800
58	SF	20160212	1036
59	SF	20160213	1516
60	SF	20160214	1918
61	CME	20160305	1900
62	CME	20160320	1400
63	CME	20160414	0900
64	CME	20160417	0300
65	SF	20160723	0146
66	SF	20160724	0609
67	CME	20160802	1400
68	SF	20160807	1437
69	CME	20161013	0600
70	CME	20161104	1800
71	CME	20161110	0000
72	SF	20161129	1719
73	SF	20170401	2135
74	CME	20170409	0000
75	CME	20170414	0000
76	CME	20170527	2200
77	SF	20170703	1537
78	SF	20170709	0309
79	SF	20170714	0135
80	SEP	20170714	0900
81	CME	20170822	0400

82	SF	20170904	0536
83	SEP	20170905	0040
84	SF	20170910	1535
85	SEP	20170910	1645
86	SF	20171020	2310
87	CME	20171225	0000
88	CME	20180309	2200
89	CME	20180513	0600
90	CME	20180606	1100
91	CME	20180625	0800
92	CME	20180630	2000
93	CME	20180710	1200
94	CME	20180825	1200
95	CME	20180923	0400
96	CME	20190507	2200
97	CME	20190511	0600
98	CME	20190514	0600
99	CME	20190516	2300
100	CME	20190527	0400
101	CME	20191029	2000
102	CME	20191102	2100
103	CME	20191111	1000

As seen from Fig.A, we choose 24-hour duration to define SWEs affected flights and quiet time flights to avoid the daily periodicity of the flight delays. Some flights data are not used if they are not in an entire day (the green area). It should be noted that the overlapping SWEs (Case B) are not counted in our analyses except the SEPs. In addition, any canceled flight is not used either. Moreover, unlike the SFs and SEPs that propagate very fast, it will take ~30 minutes for a CME from being observed (at L1 point) to the magnetosphere, so an additional 30 minutes' compensation is applied to all CMEs. Finally, all the time are converted to the local time (UTC+8) in data processing.

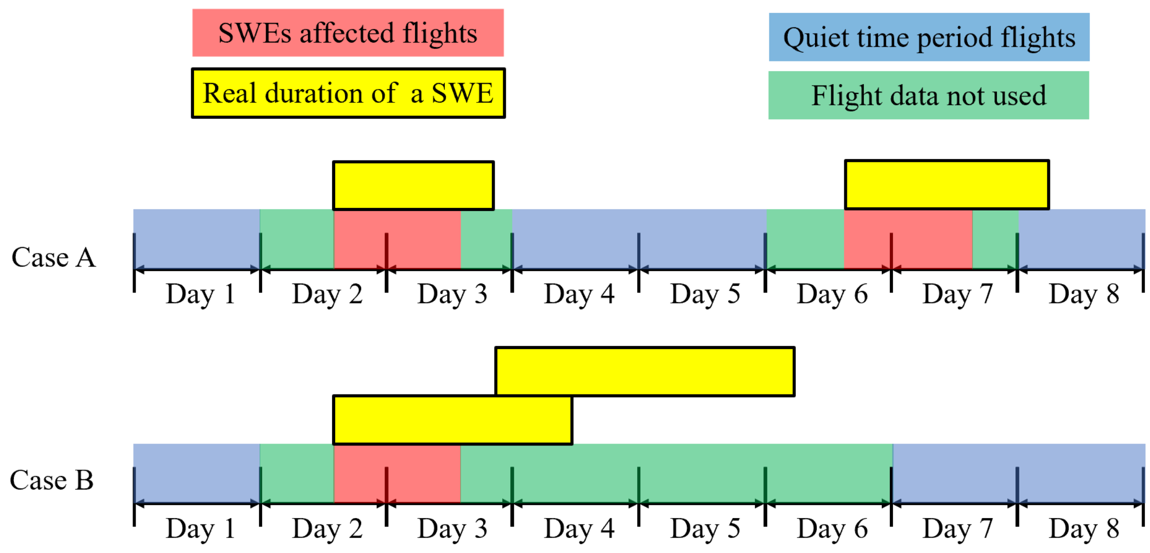

Fig.A. The definition of SWEs affected flights and quiet time flights.

The flight delay time discussed in this paper is the difference between the actual and scheduled gate in time. The number of flight records analyzed in QTPs, SWEs, SFs, CMEs and SEPs are 3426757, 271316, 134556, 115951 and 20809 respectively, while 1635280 flight records are filtered out.

The Dst index is obtained from the International Service of Geomagnetic Indices:
http://isgi.unistra.fr/data_download.php

The TEC data is obtained from the Madrigal database:
http://cedar.openmadrigal.org/index.html

The foF2 data is obtained from the Meridian Project Data Center:
https://data.meridianproject.ac.cn/

The TEC and foF2 data depend on the location and time. The location of the arrival airport and the arrival time are chosen to match each flight record. The TEC data is calculated by an automated software package to process GPS data based on a network of worldwide GPS receivers, and the vertical TEC data are adopted here. The spatial resolution of TEC data is 1° (latitude) × 1° (longitude) and the temporal resolution is 5 minutes. Since the TEC data usually have data gaps, we averaged the TEC data over a square 5°(latitude)×5°(longitude) region for each airport.

The time resolution of the foF2 is 15 minutes. The regional foF2 data also have many data gaps as the TEC data, so we have to use the data from the other nearest stations to match each airport if the local data in unavailable. The data from Mohe station (E122°22′12.00″, N53°30′0.00″) is used to match PEK, Wuhan station (E114°36′36.00″, N30°31′48.00″) is used to match SHA and PVG, Hainan station (E109°7′58.80″, N19°31′33.60″) is used to match SZX and CAN.
